# Energy use strategies and implications for fire risk amongst low-income households

**DOI:** 10.4102/jamba.v12i1.890

**Published:** 2020-12-14

**Authors:** Alberto P.M. Francioli

**Affiliations:** 1 Research Alliance for Disasters and Risk Reduction, Faculty of Arts and Social Sciences, Stellenbosch University, Stellenbosch, South Africa

**Keywords:** energy, electricity, dwelling fire, risk, low-income residential area, energy stacking

## Abstract

Despite near universal access to electricity in Cape Town, usage of informal electrical connections and nonelectric energy sources remains high and pose significant fire risk to such households. This research set out to examine the energy sources being utilised by low-income households in Lwandle, Nomzamo and Asanda Village to understand the factors that influence these energy use choices and what implications these energy choices have for fire risk. This research utilised a mixture of qualitative and quantitative data collection methods including focus group sessions with residents and a household survey to collect information on household energy use strategies, perceptions of safety and accessibility of energy sources and experiences of energy-related fires from residents residing in different types of dwellings. The research observed that despite high access to electricity, household utilisation is constrained by economic and physical factors. Consequently, they are forced to resort to employing an energy stacking approach, alternating between electric and nonelectric energy sources, which include usage of cheaper yet potentially hazardous energy sources such as paraffin (kerosene), candles, firewood, coal and gas to meet their daily energy needs. A potential consequence of this energy stacking approach employed by households to meet their energy needs is that the majority of households continue to face the risk of a dwelling fire caused by nonelectric energy sources. Whereas nonelectric energy sources were both perceived and experienced by residents as the main cause of dwelling fires in the study site, electricity was found to contribute to a number of dwelling fires, with a slight increase in the number of fires caused by electric sources observed over the last few years.

## Introduction

Fires have been a constant problem amongst residents of low-income residential areas such as in the City of Cape Town (Pharoah et al. 2013). A common cause of these dwelling fires have been attributed to the usage of unsafe and potentially hazardous forms of energy used for daily activities such as candles, for lighting, paraffin for cooking and boiling water and firewood for heating of dwellings. It has often been prescribed that key to curbing dwelling fires amongst low-income residential areas is to increase people’s access to more modern and safe forms of energy such as electricity (Albertyn et al. 2012; Spalding-Fecher 2005).

During the last 20 years, the City of Cape Town has endeavoured to address energy inequality by improving access to electrical infrastructure and services to impoverished and low-income communities across the municipality (CoCT 2015). Yet despite near-universal access to electricity in these areas, dwelling fires remain a frequent occurrence as it is believed that many low-income households continue to utilise dangerous nonelectric energy sources, as well as increasing reports of fires caused by faulty or informal electric connections (Pharoah et al. 2013; Swart & Bredenkamp 2012; Western Cape Government 2017).

Unfortunately, there is relatively limited information about actual energy use strategies employed by such households, what factors influence and determine such energy choices and the implications that these energy use strategies have for the incidence of dwelling fires. Therefore, this research set out to explore and examine what energy sources are being utilised by low-income households and their implications for fire risk in these households.

This article presents some of the findings of a study investigating energy use strategies amongst residents of low-income households in Lwandle, Nomzamo and Asanda Village communities in the City of Cape Town, the factors influencing these energy use choices and the implications such energy usage has upon dwelling fire risk.

## Background on energy and fire risk in South Africa

Since the 1990s, the South African government has endeavoured to increase access to modern energy services to its citizens, particularly those who are poor and were previously disadvantaged by the policies of the apartheid government (Huchzermeyer & Karam 2006; Tredoux 2009). By 2013, it was estimated in the South African General Household Survey that the South African government had provided over 5.7 million households with physical access to electricity across the country, increasing the proportion of the population with access to electrical services from 36% to 88% since the end of apartheid (RSA 2014). Electricity has also been made largely available to informal households such as informal settlement dwellings (ISDs) and backyard dwellings (BYDs) through informal connections which are accessed from a neighbouring electrified dwelling or illegally siphoned from electrical infrastructure such as electric transformers or power lines (Franks & Prasad 2014; Kovacic et al. 2016; Smith 2005; Zweig 2015).

**FIGURE 1:**
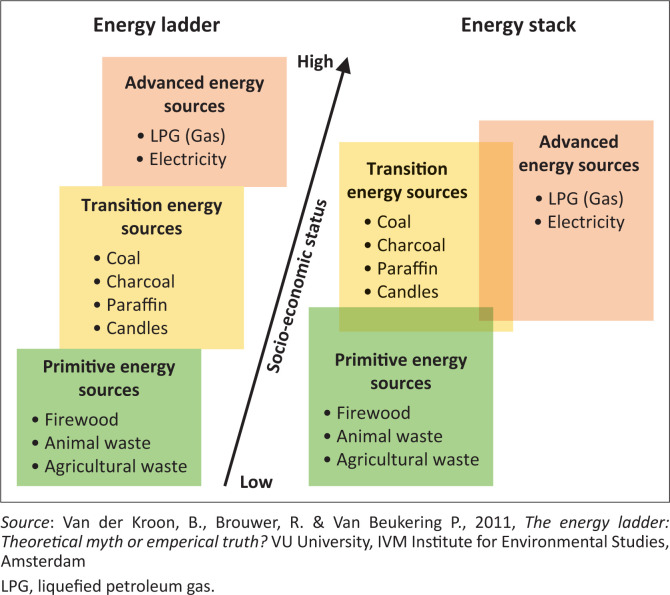
Energy transition process: Energy ladder model versus energy stacking model.

According to the City of Cape Town State of Energy (CoCT SoE) 2015 Report, it was estimated that over 94% of all households across dwelling types have access to electricity through either formal or informal connections – one of the highest rates of electrical access in the country (CoCT 2015). However, despite high access to electricity via formal or informal infrastructure, low-income households in the City of Cape Town still appear to continue to utilise a range of energy sources other than electricity to meet their daily energy needs. Energy sources such as paraffin, candles and biofuels such as fire wood and coal are still widely utilised and sought after by low-income households (Panday & Mafu 2007; Swart & Bredenkamp 2012; Winkler et al. 2011).It has been observed that such households may interchange between electric and nonelectric energy sources for particular activities, such as switching between paraffin and electricity for cooking and candles and electricity for lighting. This employment of a mixture of energy sources by such households to meet energy needs is commonly referred to as an ‘energy stacking approach’ (Van der Kroon et al. 2011) as illustrated in Figure 1.

This approach has been observed across the country, as well as amongst other low-income communities internationally, to be utilised by households when access to electricity is not assured (RSA 2012; Swart & Bredenkamp 2012; Winkler et al. 2011). For example, Lloyd (2014a, 2014b) observed that many low-income households struggle to afford adequate electricity or the necessary appliances to meet their daily energy needs because of low, irregular and or seasonal income flows. Thereby, they resort to utilising nonelectric energy sources and their associated appliances such as candles and paraffin, which are relatively cheaper and very accessible to purchase from local stores or even borrow from neighbours.

Unfortunately, the utilisation of these nonelectric flame-based energy sources places households at significant risk of experiencing dwelling fires. Dwelling fires are an ongoing challenge for urban residents across South Africa, particular those residing in informally built dwellings (Pharoah 2009; Pharoah et al. 2013). In Cape Town, over 16 000 residential fires were reported by emergency services between 2009 and 2016, of which 7605 (47%) were in informal dwellings (Western Cape Government 2017). Numerous community risk assessments, undertaken by risk researchers found that residents of such communities exist in a constant state of fear of losing their dwelling, possessions and even incur injury or death from dwelling fires (DiMP 2010, 2011, 2012). These fires are often attributed to accidents involving the usage of nonelectric energy sources and appliances such as candles, paraffin stoves and burning biofuels (Truran 2009; Western Cape Government 2015). According to research by Swart and Bredenkamp (2012) approximately a third of all informal dwelling fires in South Africa are caused by candle-related accidents, that is, being knocked over or left unattended (Greeff & Lawrence 2012; Swart & Bredenkamp 2012). The Paraffin Safety Association of South Africa (PASASA) estimated about 56% of dwelling fires were attributed to paraffin-related ignitions in South Africa (Lloyd 2012; Swart & Bredenkamp 2012). Dwelling fires have been known to start from accidentally knocking over paraffin stoves or leaving them unattended for too long (Kimemia & Van Niekerk 2017; Rosenberg 2013).

Whereas nonelectric energy sources are frequently blamed for fires, it appears that with increasing access to electricity, more fires caused by old and faulty electronic appliances, exposed informal wiring and irresponsible or negligent usage of such appliances are being reported (Albertyn et al. 2012; Lemaire 2015; Rosenberg 2013). The City of Cape Town’s data on fire incidents between 2009 and 2015 reveals electric-based fires have increased by 132% amongst formal dwellings and 334.5% amongst informal dwellings (Western Cape Government 2017). It was also observed from the city’s data that the proportion of residential fires caused by electricity in Cape Town had increased from 10.9% in 2009 to almost 25% of all residential fires by the end of 2015 (Western Cape Government 2017).

## Overview of methodology

### Study site location and selection

**FIGURE 2:**
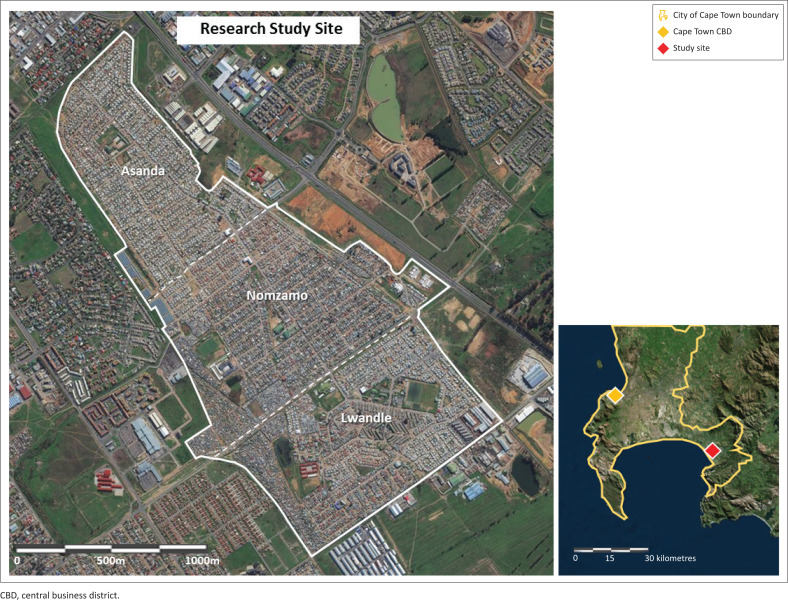
Map of the research study site; Nomzamo, Lwandle and Asanda Village.

This research took place amongst the residential suburbs of Lwandle, Nomzamo and Asanda Village, close to Somerset West and Strand within the City of Cape Town Metropolitan area, as shown in Figure 2. According to 2011 census data the majority of households in the site earn a low-income (i.e. under R5000.00 per month) (RSA 2012). The site has a diversity of residential dwelling types, including government built formal low-cost ‘RDP’ housing, often accompanied by one or more BYDs and stand-alone ‘shacks’ (ISDs) in the informal settlement in the southern region of the study site. These dwellings have a mixture of formal electrical access (legally installed infrastructure and electricity meters) and informally constructed illegal connections, which tap electricity from neighbouring electrified dwellings or from electric infrastructure such as power lines and substations.

### Data collection methodology

A mixed methods approach was utilised to collect both qualitative and quantitative data for this research. The qualitative primary data were gathered through:

- Consultation and interviews with various key stakeholders, such as personnel from Helderberg Disaster Management, Strand Fire Station, Electricity Department, Local Ward Councillors and local museum staff, to attain a general community-based perspective of the energy-related issues experienced by the residents of the study site.
- Carrying out 60 door-to-door semi-structured interviews with households based in the three main dwelling types (20 Formal dwellings, ISDs and BYDs each) to identify the types of energy different households utilised, whether they perceived them as a fire hazard and whether they have experienced a dwelling fire initiated by an energy source in their dwelling. Households were selected through a random convenience sample depending upon residents’ willingness to participate in the interview.
- Holding eight focus group sessions to discuss and debate issues with approximately 100 residents. These participatory focus groups sought to identify significant fire events, energy use preferences for particular activities, the positive and negative attributes of energy sources and strategies to reduce the risk of fires.

**FIGURE 3:**
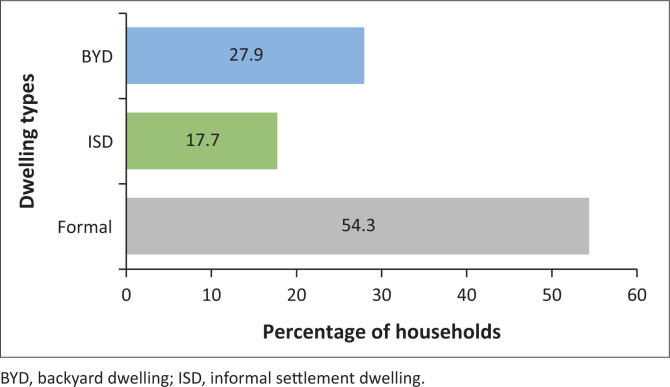
Percentages of households surveyed by dwelling type (*n* = 530).

Primary quantitative data were captured through the distribution of 530 household surveys by the author and eight assistants over a period of 18 days. Of the 530 households that participated in the household survey, as shown in Figure 3, approximately 54.3% of households surveyed resided in formal dwellings (these included both RDP and hostel dwellings), 27.9% within BYDs, 17.7% within ISDs.

The survey was predominantly comprised of pre-coded closed-ended questions and it also included some open-ended questions, allowing participants to provide their own in-depth explanation and feelings on a particular issue.

### Ethical considerations

The author confirmed that ethical clearance was not required for the study.

## Results and analysis

### Energy stacking approach employed amongst different households

The data found that 517 (97.6%) of the 530 households surveyed across the study site have access to electricity through some form or another. Formal households acquired electricity through ‘formal’ electrical infrastructure built within their homes, connecting them to the national electrical grid. Those residing in informal dwellings such as BYDs and ISDs acquired their electricity through ‘informal’ electrical connections in the form of extension cords or makeshift wiring. The BYDs acquire electricity from connections between themselves and the formal dwelling whose property they reside upon, in which they pay the formal dwelling for this access pay. Informal settlement dwellings often follow a similar system of access, in which ISDs situated closest to formal housing areas will pay for access to their electrical connections. In other instances, ISDs acquire electricity by siphoning power from formal electrical infrastructure, such as nearby power lines and electricity substations illegally. These connected ISDs then disburse their acquired electricity to neighbouring ISDs, who do the same, creating a vast informal web-like network of informally constructed cabling and wiring shared amongst hundreds of ISDs.

Electricity was the most preferred/desired energy source as it was perceived as making residents’ lives easier, especially for activities such as cooking, boiling water and heating because all they had to do was switch on an appliance, rather than try to ignite a flammable energy source and keep watch over it to prevent it from setting something alight.

However, across dwelling types, many households perceive electricity and electric appliances to be very expensive, particularly for households with low per capita income, seasonal or irregular employment, large households with a single breadwinner and those reliant on social grants such as pensions. Many BYD- and ISD-based residents complained that landlords and those distributing electricity charged very high rates, which were difficult to afford. Similar observations were made by Lloyd (2014a, 2014b) in several studies in which low-income households struggle to afford adequate electricity or the necessary appliances such as microwaves, electric stoves, etc., to meet their daily energy needs.

Another issue constraining usage of electricity amongst dwellings was that of quality of connection. While formal households generally have high quality professional placed connections and wiring, informal connections used in BYDs and ISDs tend to be of poorer quality, and are less reliable and safe. They are often restricted from using too many appliances in their dwelling to avoid placing pressure on their connections and causing power outages or damage to the connections. This is particularly a problem amongst ISDs in which a single dwelling may cause a ‘trip’ which may cut power to dozens of other dwellings on the same network.

**FIGURE 4:**
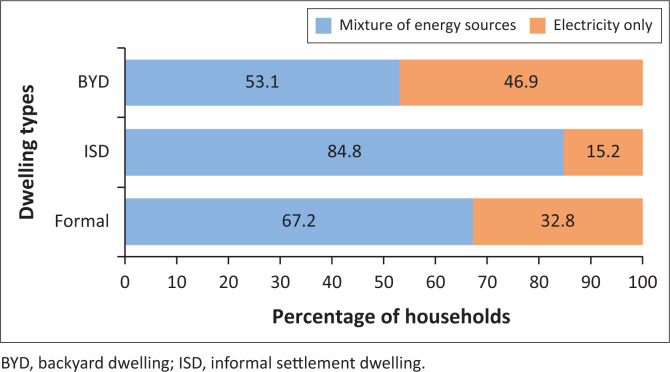
Proportion of households utilising a mixture of electrical and nonelectrical energy sources (*n* = 530).

**FIGURE 5:**
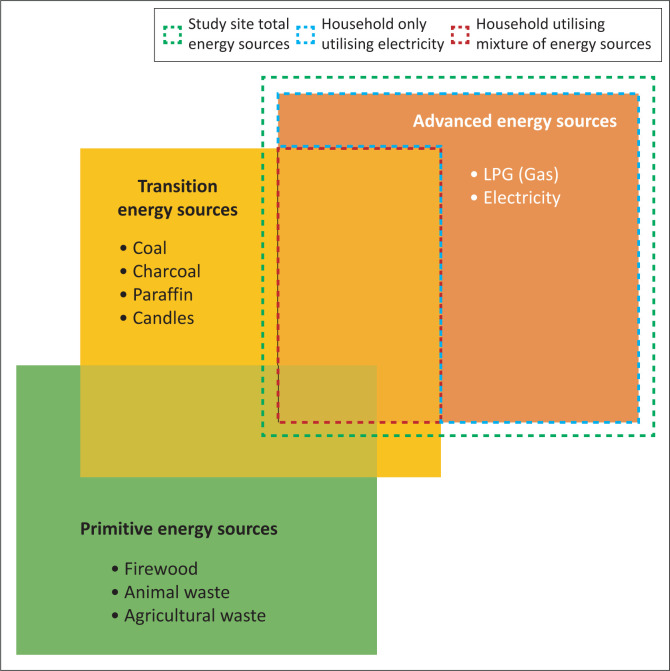
Location of study site households within the energy stacking model.

As a result of inadequate financial resources and/or poor quality of connections to electricity, approximately two-thirds (67.2%) of households in the study site showed evidence of employing an energy stacking approach (as can be viewed per dwelling type in Figure 4) utilising a mixture of electricity and other nonelectric energy sources, such as paraffin, candles, gas, firewood and coal, to meet their household’s energy needs (as illustrated in Figure 5 situating households within the energy stacking model). This finding mirrors observations by Van der Kroon et al. (2011) and Uhunamure, Nthaduleni and Agnes (2016) who observed that energy use in low-income households does not tend to correspond to the energy ladder approach and the implication implied assumption being that people will adopt more advanced sources when they become accessible, but will continue to utilise a mixture of primitive, transitional and advanced energy sources to meet their needs.

Paraffin (also known as kerosene) has remained a commonly utilised energy source amongst households surveyed and a popular alternative to electricity, particularly amongst ISDs. It is a highly versatile energy source, used for activities such as cooking, boiling water and spatial heating. Paraffin is particularly popular during the winter, as it serves to provide energy for cooking and spatial heating simultaneously from the same, whereas electricity users would use separate appliances to perform such function separately. It is also highly accessible within the study site, being sold from local ‘spaza’ shops and corner stores, in which residents can purchase literally ‘by the cup’, as it can be borrowed and shared amongst neighbours. The ability to borrow, share and transport paraffin easily among households, as observed in the research, mirrors an observation by Lloyd (2014b) who speaks of paraffin as a “social fuel”, hence contributing to its popularity and high usage among low-income households.

Candles are still employed regularly by some households for lighting because they, like paraffin, are deemed inexpensive and highly accessible. Almost all houses keep candles as a backup, because of frequent power cuts and trips caused by over usage of electrical appliances. Firewood and coal tend to be reserved for cooking for special occasions such as family gatherings, holidays, traditional ceremonial events and the like. A handful of households operate small businesses where they sell meat cooked on an ‘mbawula’ (a makeshift fireplace often constructed from a steel drum). Several residents reported that a strategy of keeping warm amongst some households, particularly residents of ISDs, was to bring in hot coals or burning logs from a fire into their dwellings. Interestingly gas was found to be rarely utilised amongst households. Despite being perceived as very efficient my majority of residents, they also saw it as a high fire risk, fearing a gas leak may cause an explosion.

An unexpected finding was the observation that residents of BYDs are less likely to resort to energy stacking approach than that of residents of formal dwellings, that is, they would utilise electricity more frequently and for more activities than most formal dwellers. A key reason was that BYD households had fewer members and fewer electrical devices, hence could use it more freely than larger households typically found in formal dwellings. It was also noted that residents of formal dwellings felt more secure whilst utilising flammable energy sources in their dwellings as they were more spacious and comprised of nonflammable materials (i.e. tiled floors and brick walls) which they believed reduced risk of dwelling fires, unlike cramped wooden shacks.

The observations of households’ use of an energy stacking approach correspond with the 2012 energy survey for the residential sector by the Department of Energy (DoE). The DoE (RSA 2012) survey found electricity remains the predominant energy source utilised for various household activities, most low-income urban households employ nonelectric energy sources frequently as alternatives to supplement their energy needs for cooking, lighting and spatial heating. Whilst this research and the DoE survey observed that energy stacking was prevalent amongst urban informal dwellings like BYDs and ISDs, they differed regarding mixed energy use strategies amongst formal households. Whilst the DoE survey reported a minority of formal households (38.5%) utilising an energy mix approach, this research observed higher rates of energy stacking amongst formal households in the study site (67.2%).

**FIGURE 6:**
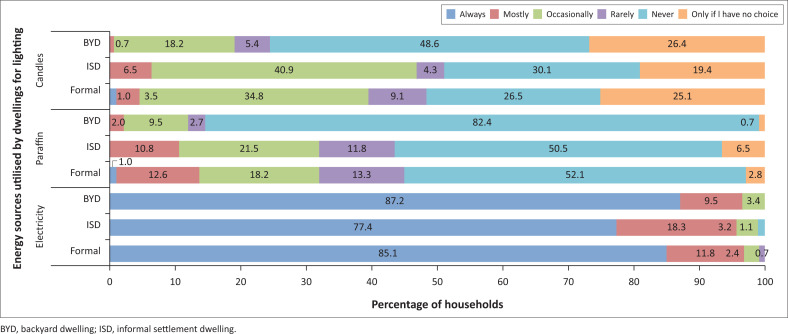
Energy sources used for lighting by households.

**FIGURE 7:**
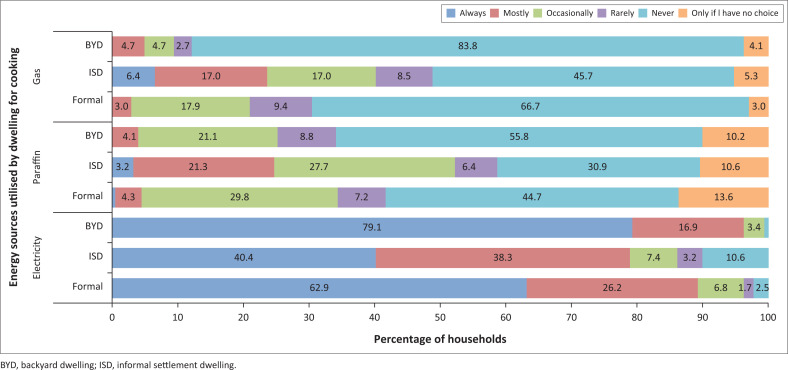
Energy sources used for cooking by households.

**FIGURE 8:**
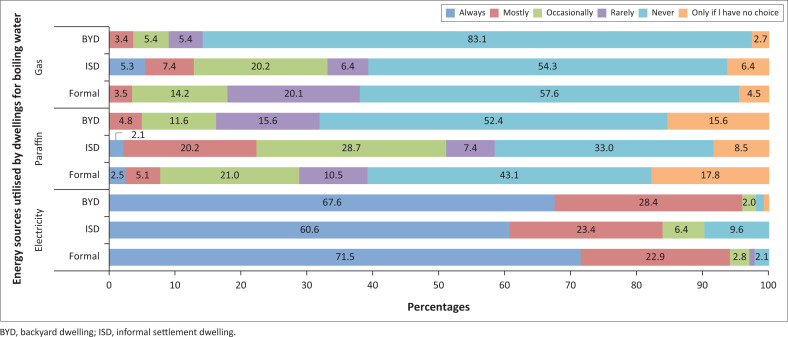
Energy sources used for boiling water by households.

**FIGURE 9:**
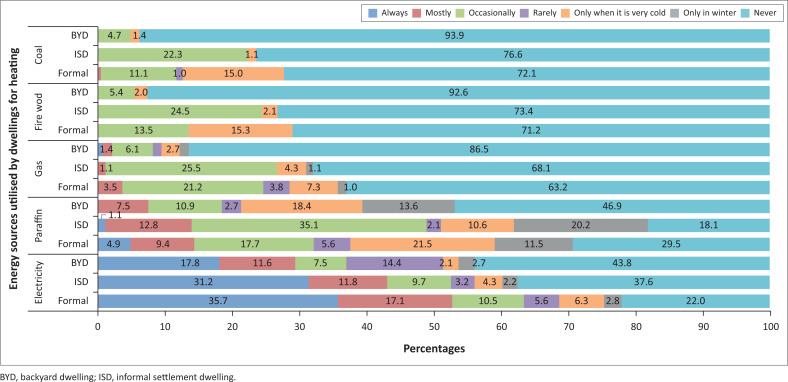
Energy sources used for heating by households.

A breakdown of the various energy sources utilised for particular activities by households residing in different dwelling types can be observed in Figures 6–9.

### Implications of energy use for household fire risk

The unfortunate consequence of the limited economic and physical inaccessibility of electricity for most households is increased exposure to nonelectric flammable energy sources, which in turn increases their risk of experiencing dwelling fires. According to the survey, 24.3% of households have experienced at least one fire whilst residing in the study site. Majority of dwelling fires experienced by households were relatively small and were quickly put out by the residents, only causing minor damage and injuries. Majority of residents stated that they did not bother to report these incidences to the local fire department because they believed it was unnecessary or a waste of their time and ‘airtime’. However, a handful of residents interviewed stated that they survived fires that had destroyed their dwellings entirely, forcing them to rebuild their homes. It was observed that most of these dwelling fires recorded have been caused by nonelectric energy sources, most commonly paraffin and candles. Several households reported paraffin stoves spouting out flames or as commonly referred to as ‘exploding’, causing instantaneous damage and a fire that is almost impossible to put out. Such ‘explosions’ can occur because of either contaminated fuel or faulty, poor-quality or worn out appliances. This corresponded with data gathered by the City of Cape Town’s Fire and Rescue Services in which they identified inexpensive but substandard quality paraffin stoves as a major culprit for dwelling fires across Western Cape settlements (Western Cape Government 2017).

**FIGURE 10:**
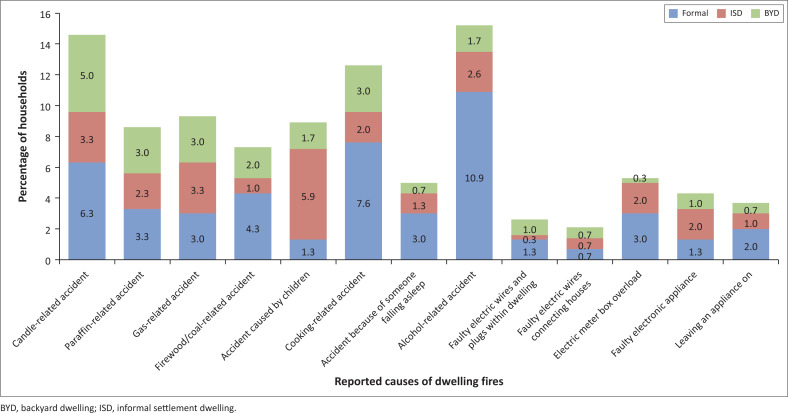
Reported causes of fires by households who have experienced one or more fire incidences.

However, it is interesting to note that the majority of fires caused by nonelectrical energy sources were not related to problems with a device, such as a malfunctioning paraffin stove. As will be seen in the list of reported causes of fire illustrated in Figure 10, the majority appear to have occurred because of accidents caused by negligent and irresponsible human behaviour whilst utilising such an energy source. Truran (2009:9) believed that whilst particular energy sources can be viewed as hazards that may cause fire, the real ‘danger is not so much paraffin per se but rather the unsafe system of paraffin use’. Participants of focus groups often identified intoxication as a major factor driving fire risk, offering numerous examples of people attempting to operate paraffin stoves whilst inebriated or passing out whilst candles are still lit. Other research has linked causes of dwelling fires to drunken behaviour, such as people returning home from a night of drinking attempt to either light a candle or cook in their inebriated state or they fall asleep leaving a flame unattended as they fall asleep (Harte, Childs & Hastings 2009; Pharoah 2012; Western Cape Government 2016). Other residents claimed that breathing in paraffin or firewood emissions/smoke could make people unwell and disoriented, thus increasing the risk of an accident such as knocking over an operating paraffin stove. Another major cause of dwelling fires identified during interviews was related to children being left unattended with something flammable. Several residents retold incidents in which young children caused fires whilst attempting to cook food for themselves, lighting candles or by literally playing with fire (i.e. igniting matches and setting objects alight as part of a game).

Unsurprisingly, fires caused by electric sources such as faulty wiring or appliances made up a minority of reported dwelling fires by surveyed residents. This finding appeared to mirror the perceptions reported by residents that electricity is a safer energy source than other energy sources. However, despite being fewer, electric-based fires reported by residents still made up 19.21% of the reported dwelling fires in the survey. As can be seen in Figure 10, electric fires were found predominantly in formal dwellings, shortly followed by ISDs. Many formal and informal residents stated that fires are often caused because of utilising too many electric appliances at once, causing trips that damage the circuitry of appliances and wiring, resulting in sparks and igniting nearby flammable objects. One resident stated that they almost had a fire the night before being interviewed because an extension cord and plug running directly beneath their mattress had (presumably) overheated had started to burn it.

These findings have implications for fire risk in different dwelling types. It is widely assumed that the roll out of electrical infrastructure in low-income areas will assist in reducing fire risk as households shift from transitional to advanced energy sources (Albertyn et al. 2012; Louw et al. 2008). However, the findings of the research suggest that this is not the case, with the majority of households still employing a mixture of electric and nonelectric energy sources. Consequently, many households still use highly flammable and unsafe energy sources such as paraffin and candles, which place households at high risk of experiencing a dwelling fire. In addition, the research indicates that there have been a significant number of dwelling fires initiated by faulty wiring and electric appliances, suggesting the electricity too poses a fire risk.

**FIGURE 11:**
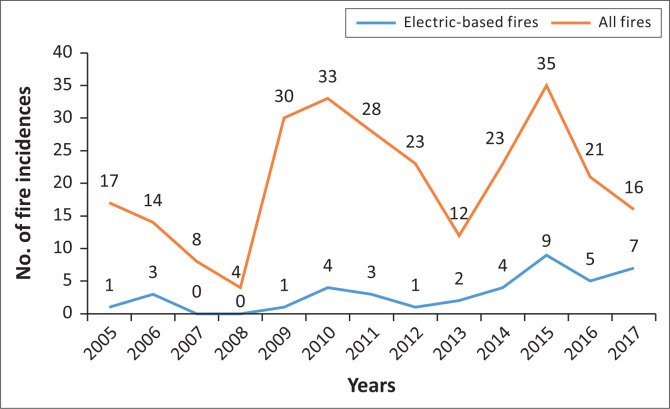
Increasing number of electric-based fires occurring in study site.

Whilst there were only a few electric-based fire incidents recorded, there was a slight increase in the number of such incidents over the years (as shown in Figure 11). It was found that in 2013, 2015 and (within the first 5 months of) 2017, electric-based fires made up approximately a third of all fires in those years, which could possibly indicate a potential rise in the frequency of electricity-related fires in the study site. This corresponds with data collected by the City of Cape Town in which electric-based fires have been on the rise across informal and formal dwellings (Western Cape Government 2017).

### Combatting household fire risks through personalised risk reduction initiatives

The majority of residents of the study site recognised that the nonelectric energy sources they utilised are potential fire hazards, threatening to cause a fire because of an accident, irresponsible usage or an appliance fault. Consequently, many households have attempted to reduce their usage of such energy sources, employing them as little as possible and using electricity in its stead, in order to reduce the household fire risks.

However, for many households, reducing the usage of nonelectric energy sources to reduce their risk to fire is not an option. Many households in the study site are constrained by limited financial resources and poor-quality electrical connections, thus forcing them to resort to alternative nonelectric energy sources.

Although most households have little option other than to utilise these potentially hazardous sources, engagement with residents identified several specific measures employed by households to attempt to mitigate their risks to fires, which include one or a combination of the following:

- ***Using a minimum of electrical appliances simultaneously to avoid overheating and sparks:*** Many households, especially BYDs and ISDs stated that one should be careful not to use too many electronic appliances simultaneous as it increases the risk of causing plugs and wires to overheat, the electricity to trip and damage to the meter box. Consequently, many households try to keep the number of electronic appliances being used simultaneously to a minimum, especially whilst cooking with electronic appliances, which consume a lot of electricity.
- ***Check and maintain all appliances and connections***: Many households, especially BYDs and ISDs, mentioned they would frequently check on and maintain their electrical connections and appliances. This behaviour included applying protective tape around loose wires and fixing and replacing broken wires and plugs. They also checked and maintained nonelectric appliances too, such as gas heaters and paraffin stoves. Several households stated that cleaning paraffin stoves frequently made them more reliable, less prone to spurting out flames or ‘exploding’.
- ***Keep away from flammable objects***: The majority if not all households try to keep any flammable objects such as clothing, curtains and furniture away from energy-based appliances, in particular those using nonelectric sources such as gas and paraffin stoves. Amongst BYDs and ISDs, people tried to ensure that wires do not run beneath beds and furniture and clothes. Most households try to position wiring on the ceiling to avoid contact.
- ***Switch off and/or disconnect:*** A commonly employed approach used across all households was to ensure all electrical appliances are properly switched off when not in use, especially when no one is in the house. Amongst many BYDs and ISDs, residents are able to disconnect their whole dwelling from their source of electricity. For BYDs, many residents, before leaving the dwelling, would unplug themselves or inform their landlord so that they could unplug them. Similarly, residents in ISDs disconnect themselves by detaching the wires that carry the electricity to the dwelling when necessary.
- ***Proper ventilation to reduce accidents because of adverse symptoms of emissions:*** An issue raised by residents was that breathing in paraffin or firewood emissions/smoke could make people unwell and disoriented, thus increasing the risk of causing an accident such as knocking over an operating paraffin stove. Consequently, several households iterated the importance of properly ventilating their dwellings to ensure the dwelling is not inundated with fumes and thereby reduce the risk of becoming unwell and causing an accident.
- ***Safety during power cuts***: If electricity is cut because of a power trip, load shedding or because of running out of electricity, many households ensure that all electric appliances are switched off, especially if they were switched on during the power cut. This behaviour is to ensure that if the power comes back whilst the household members are out, the appliances do not turn on and continue operating unsupervised.
- ***Protecting and educating children:*** Many households prioritised keeping candles, matches, paraffin and electric stoves out of reach of children – this also included not leaving children alone in the house or unsupervised. For households that used paraffin stoves for heating in winter and candles for emergencies, they suggested hiding or locking up these things so that children could not access them. Similarly, households stressed the importance of teaching children from a young age not to play with flammable appliances and forbidding children from using candles and flammable appliances unless supervised and teaching children how to use these safely.
- ***Safety during cooking:*** Cooking was noted by many as a potentially dangerous activity. Households reported that there should always be someone supervising cooking. One respondent noted that he does not cook when he is drunk or hung-over, in case he passes out and leaves the stove on.
- ***Community vigilance:*** In informal residential areas of the study site, a form of risk reduction comes from strong community ties and vigilance for fire. As a result of the high risk of fires occurring amongst these areas and the risk of fire spreading quickly to surrounding dwellings, community members are often on the lookout for signs of fire. Therefore, neighbours often watch out for one another to give warning if a fire breaks out and give aid to fight the fire if necessary.

## Practical managerial implications and recommendations

Whilst most of the households in the study site have physical access to electricity, this research finds that having access does not mean that all households can use it. The prohibitive cost of electricity and limited physical accessibility prevents most households from using electricity to meet all or even part of their energy needs and are forced to employ hazardous nonelectric energy sources. This also challenges the assumption

that the roll out of electrical infrastructure in low-income areas will assist in reducing fire risk as households shift from transitional to advanced energy sources (Albertyn et al. 2012; Louw et al. 2008). However, the findings of the research suggest this is not the case, with 62.7% of households still employing a mixture of electric and nonelectric energy sources. Consequently, there remains high usage of highly flammable and unsafe energy sources such as paraffin and candles, which place households at high risk of experiencing a dwelling fire. In addition, the research indicates that there have been a significant number of dwelling fires initiated by faulty wiring and electric appliances, suggesting that the electricity too poses a fire risk.

This highlights a need for continued and holistic measure to reduce the risk of fires in low-income areas. Whilst current efforts to reduce risk tend to focus in informal settlements, the research suggests that the potential for fires in formal housing areas caused by particular energy sources needs to be better understood, and that initiatives to prevent fires need to extend to formal housing areas and not just informal settlements. A particular strategy advocated by the Western Cape fire services that may significantly reduce fire risk in both formal and informal dwellings is to promote legislation to regulate the design and quality of paraffin stoves, to ensure that substandard and inferior quality devices are barred from being sold to households (Western Cape Government 2015).

It is also essential that campaigns to raise awareness about fire-prevention include education on the dangers posed by electricity and how to utilise it safely. A prominent area of action identified by fire services has been to reduce human and behavioural risks through improved educational and awareness campaigns to promote safe energy use practices, particularly amongst children (Western Cape Government 2015). Another intervention advocated by disaster management and fire services in the Western Cape is to invest in household warning systems, particularly smoke alarms to provide early detection of ignition of accidental fires, providing opportunity to prevent a disaster or at least mitigate the impacts (Western Cape Government 2015, 2016). The proposed implementation of such alarms amongst low-income dwellings may be crucial considering the prevalent usage of unsafe energy sources such as paraffin and candles and even fires caused by electric wiring and appliances.

## Conclusion

In this research, low-income households across Lwandle, Nomzamo and Asanda Village were observed using a wide diversity of energy sources other than electricity to meet their energy needs because of issues of financial and infrastructural constraints.

A potential consequence of the energy stacking approach is that the majority of households continue to face the risk of a dwelling fire caused by nonelectric energy sources. The frequent use of nonelectric energy sources is perceived by residents as a major contributor to fires in the study site. The research shows that the majority of dwelling fires experienced by residents in the study site are caused by nonelectric energy sources, particularly candles and paraffin. The danger posed by these energy sources is strongly linked to human behaviour such as drinking and children knocking over or playing with flammable energy sources. Although electricity is regarded as a safer source of energy, the research found that a number of fires have been caused by faulty electrical wires, appliances, informal connections and overloading of plugs and electrical systems. Although the blame for dwelling fires has been predominantly placed upon nonelectrical energy sources such as paraffin, it appears that the number of electric-based fires is on the rise and becoming a more common driver for such risk.

This research highlights the need for increased efforts to reduce fires not only amongst households-based informal settlements but amongst formal dwellings as well as BYDs. Initiatives to reduce fire risk should not be solely focused upon addressing hazardous energy sources present in households but also include campaigns to raise awareness and promote safe usage of both electric and nonelectric energy sources. Another initiative could be to invest in technologies such as smoke alarms to provide early detection and possible prevention of such dwelling fires from occurring.
